# Obsessive-compulsive symptoms in two patients with strategic basal ganglia lesions

**DOI:** 10.1038/s41380-022-01853-8

**Published:** 2022-11-10

**Authors:** Dominique Endres, Katharina von Zedtwitz, Horst Urbach, Rick Dersch, Kimon Runge, Bernd Feige, Kathrin Nickel, Miriam A. Schiele, Harald Prüss, Katharina Domschke, Marco Reisert, Volker A. Coenen

**Affiliations:** 1 Department of Psychiatry and Psychotherapy, Medical Center—University of Freiburg, Faculty of Medicine, University of Freiburg, Freiburg, Germany; 2 Department of Neuroradiology, Medical Center—University of Freiburg, Faculty of Medicine, University of Freiburg, Freiburg, Germany; 3 Department of Neurology and Neurophysiology, Medical Center—University of Freiburg, Faculty of Medicine, University of Freiburg, Freiburg, Germany; 4 Department of Neurology and Experimental Neurology, Charité—Universitätsmedizin, Berlin, Germany; 5 German Center for Neurodegenerative Diseases (DZNE), Berlin, Germany; 6 Center for Basics in Neuromodulation, Medical Center—University of Freiburg, Faculty of Medicine, University of Freiburg, Freiburg, Germany; 7 Department of Stereotactic and Functional Neurosurgery, Medical Center—University of Freiburg, Faculty of Medicine, University of Freiburg, Freiburg, Germany; 8 Department of Diagnostic and Interventional Radiology, Medical Physics, Medical Center—University of Freiburg, Faculty of Medicine, University of Freiburg, Freiburg, Germany; 9 Center for Deep Brain Stimulation, University of Freiburg, Freiburg, Germany

**Keywords:** Psychiatric disorders, Neuroscience

## To the Editor:

Obsessive-compulsive disorder (OCD) is a severe mental condition neuroanatomically characterized by cortico-striato-thalamo-cortical (CSTC) loop dysfunction [1], but also involves alterations in other networks [1, 2] as well as molecular and genetic changes [3–5]. Strategic lesions can result in distinct CSTC loop dysfunctions [6, 7]. This work presents two well-studied OCD patients with strategic basal ganglia pathologies which have been classified in vivo.

The diagnostic work-up in both patients was performed according to an established diagnostic protocol [8, 9]. The magnetic resonance imaging (MRI) analysis included diffusion tensor imaging (DTI) tractography along the brain lesions [10]. The electroencephalography (EEG) was analyzed using independent component analysis (ICA) [11]. Both patients gave written informed consent for publication within a cumulative case study.

Cases 1 and 2 were hospitalized for exacerbations of obsessive-compulsive symptoms (OCS).

**Fig. 1:**
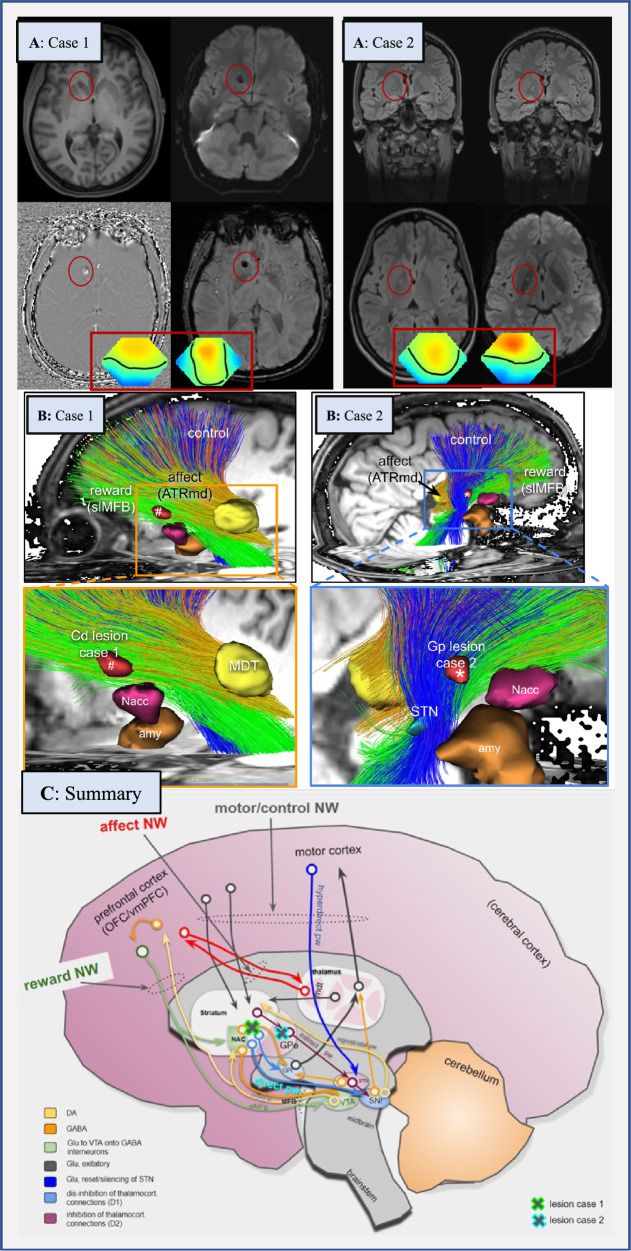
Neuroimaging findings in both patients (A, B) and conceptual considerations (C). A: Case 1 shows a lesion in the right caudate nucleus with a size of 8.9 × 5 mm. The lesion was classified as a probable microbleeding, but calcification as a late consequence of microbleeding could not be definitively ruled out. Case 2 presents with a FLAIR-hyperintense lesion in the globus pallidus on the right (defect with a gliotic rim in the direction of a lenticulostriate vessel). Because Case 2 had febrile seizures during her first decade, and since then chronic obsessive-compulsive disorder (OCD), a post-inflammatory origin could be plausible. Framed below in red are the topographies of the prominent theta electroencephalography (EEG) components for the two patients. In both cases the pronounced frontocentral theta activity is suggestive of sleepiness. Topographies are against common average reference with standard orientation (top = front), positive excursions in red, negative in blue. Zero potential is indicated by a black line. Spontaneous EEG was recorded during a 12-minute series of standard maneuvers (eyes open, eyes closed, and hyperventilation) according to the 10–20 system (21 head channels), leading to 21 independent component analysis (ICA) components. Artifact-free sections across the whole recording length were submitted to ICA training. ICA maps as well as associated EEG time courses were reviewed and components classified by their topography, reaction to the maneuvers, and spectrum. Additionally, intermittent rhythmic delta/theta activity was detected in the artifact-free sections using an automated algorithm. The software used for these analyses is a combination of the EEG data processor “avg_q” (https://github.com/berndf/avg_q) and custom python scripts. B: Case 1 has tractographic rendition of streamlines involved in strategic unilateral lesions in the inferior and anterior caudate nucleus (nucleus accumbens spared) (#, right side). Tractographic analysis of Case 2 shows a pallidal lesion (*) involving the “motor-control network” (blue fibers). C: Interplay of three involved networks (“affect, control, and reward network”) within the whole “OCD framework” (existing of the “affect, control, reward, and default-mode network” [2, 13]) at distinct network hubs (taken and altered from [13]). The locations of lesions in Cases 1 and 2 are shown as green/blue crosses. Abbreviations: Amy amygdala, ATR anterior thalamic radiation, ATRmd anterior thalamic radiation from dorsomedial thalamus, BNST bed nucleus of stria terminalis, Cd caudate nucleus, DA dopamine, EEG electroencephalography, GABA gamma-aminobutyric acid, GPe globus pallidus externus, GPi globus pallidus internus, Glu glutamate, imMFB infero-medial MFB, ICA independent component analysis, MDT mediodorsal thalamus, MFB medial forebrain bundle, NAC/Nacc nucleus accumbens, NW network, OCD obsessive-compulsive disorder, OFC orbitofrontal cortex, pw pathway, slMFB superolateral branch of the medial forebrain bundleMFB, SNr substantia nigra, STN subthalamic nucleus, VTA ventral tegmental area, vmPFC ventromedial prefrontal cortex.

Patient 1 was a 30-year-old female patient who had been suffering from obsessive thoughts and compulsive actions for approximately five years. Her symptoms had exacerbated one year prior to her hospitalization with washing compulsions and concerns about contamination (Y-BOCS score: 27; OCI-R score: 33). The diagnostic work-up identified an MRI lesion in the right caudate with a size of 8.9 mm × 5 mm, most probably a previous microbleeding (Fig. 1). The DTI tractographic rendition of streamlines involved in the strategic unilateral lesion in the right nucleus caudate showed that the crossing fibers connected the superolateral branch of the medial forebrain bundle to the mediodorsal thalamus and potentially to the bed nucleus of the stria terminalis. ICA detected frontocentral theta activity. The CSF analysis showed slightly blood-CSF barrier dysfunction with elevated albumin quotients; no antibodies against extra- or intracellular neuronal antigens could be detected [12]. The long-term blood pressure measurement was normal. Inpatient cognitive behavioral therapy (CBT) with exposure and response prevention (ERP) over approximately 10 weeks and psychopharmacological treatment with sertraline (200 mg/day; blood levels of 54 ng/ml; reference: 10–50 ng/ml) resulted in considerable improvement in OCS, with Y-BOCS decreasing from 27 to 10 (−63%) and OCI-R from 33 to 11 (−67%).

Patient 2 was a 26-year-old female patient who had suffered from mixed obsessive thoughts and compulsive actions since childhood. This patient’s OCS were dominated by washing compulsions (Y-BOCS: 16; OCI-R: 27). Her symptoms had exacerbated two years prior to her hospitalization without any identifiable trigger. The patient experienced febrile seizures during her childhood, and she had subsyndromal ADHD and autism symptomatology (not fulfilling diagnostic ICD-10 criteria) since her first decade. The diagnostic work-up identified a FLAIR-hyperintense lesion in the right globus pallidus (Fig. 1). A tractographic analysis showed the involvement of fibers from the “motor-control network”. The lesion was in her right globus pallidus externus and at the same time encroached on the “indirect pathway”. ICA detected regional slowing in form of frontocentral theta activity. CSF analysis identified isolated increased lactate levels; neuronal antibodies were negative [12]. The results of the stroke screening and lactate ischemia tests were normal (see Supplemental Table 1). Therapy with 50 mg of sertraline (blood levels of 39 ng/ml; reference 10–50 ng/ml; higher doses led to side effects and increased serum levels) and CBT with ERP did not lead to unequivocal improvement in OCS (Y-BOCS increased to 18 [+12.5%]; OCI-R reduced to 17 [−37%]).

Here, two paradigmatic OCD cases with different strategic basal ganglia lesions and diverse therapy responses are presented. ICA detected frontocentral electrophysiological changes in both patients. The DTI tractography revealed the precise localization of the lesions. In Case 1, the lesion was in the inferior part of the caudate nucleus (ventral striatum adjacent to nucleus accumbens) and the border to the anterior limb of the internal capsule, through which the anterior thalamic radiation out of the medio-dorsal thalamus, the superolateral branch of the medial forebrain bundle, and many frontopontine connections pass. Lesions in the left caudate nucleus have previously been described as associated with OCD [6, 7]. The tractography in Patient 2 showed an involvement of the “motor-control network”. Therefore, the tractographic work-up of both patients showed lesions in two distinct parts of the “OCD framework” [2, 13]. The unilateral lesion in the right caudate of Patient 1 might have disturbed the interconnection of the “reward and affect network,” with reduced dampening of the latter and consecutive exacerbation of OCS. It can be assumed that the right globus pallidus lesion in Patient 2 involved the “motor-control network”. Therefore, the motor consequences of obsessions, including emotional consequences, could have been influenced. The globus pallidus is the exit of the basal ganglia and inherits part of the indirect motor pathway. Motor actions (proceeding or stopping) can be altered via GABA-ergic projection from the nucleus accumbens to the globus pallidus internus; and a lesion in the globus pallidus externus might disturb parts of the indirect pathway as such responsible for stopping non-intended movements and actions. It could be hypothesized that the compensated motor state (no compulsive actions) might have been altered through the lesion (compulsive actions), thus possibly unmasking the OCD.

Interestingly, only Patient 1, who had an involvement of the “reward and affect network”—but not Patient 2, with involvement of the “motor-control network”—benefited clearly from the guideline-based treatment applied in both cases. The lesion in Patient 2 showed involvement of the indirect pathway which is D2 receptor dominated. The D2 receptor, moreover, is involved in decreased reinforcement learning as a side-effect of agonistic medication [14]. Therefore, in similar cases, a pharmacological therapy targeting dopaminergic transmission might be beneficial.

The changes within the “OCD framework” level do not explain the cause of the lesions. In Patient 1, microbleeding, and in Patient 2, a post-inflammatory origin was suspected.

One limitation of both case studies is that (epi)genetic and neurochemical processes were not analyzed in detail [4, 15]. Further, psychological factors [5] may also be involved, and the role of MRI lesions may be overinterpreted.

In summary, these cases show that a broad diagnostic work-up can accurately locate lesions that might lead to “OCD framework” dysfunction [1, 2, 5, 15]. Thus, the investigation of such cases could help to better understand the pathophysiology of OCD and may support biomarker-guided treatment in the future.

## Supplementary information

Supplemental Material

## Data Availability

All necessary data can be found in the paper.
