# Supplementary material for: Obsessive-compulsive symptoms in two patients with strategic basal ganglia lesions
Source: Mol Psychiatry. 2022 Nov 10;28(2):528–31. doi: 10.1038/s41380-022-01853-8 (PMC9908536; doi:10.1038/s41380-022-01853-8)
Supplement: Supplementary file 1 — Supplemental Material [file 41380_2022_1853_MOESM1_ESM.pdf]

SUPPLEMENTAL TABLE

	Case 1: Female, 30 years old	Case 2: Female, 26 years old
**Initial Y-BOCS and OCI-R scores**	27<br>33	16<br>27
**Serum antibodies and immunological markers**		
Anti-thyroid antibodies (against TPO, TG and TSH-receptor)	**Anti-TPO 78.1 IU/ml (ref. <34 IU/ml); TG-antibodies 201 IU/ml (ref. <115 IU/ml),** TSH-receptor antibodies negative	Negative
ANAs (*on HEp-2 cells*), ANCAs (*on EthOH- /formalin-fixed neutrophils*), APAs, SMA	**ANAs (HEp-2 (nucleus 1:50)) a trace homogeneous**, ANCAs /APAs negative	ANAs/ANCAs/APAs negative, **SMA trace positive**
Complement factors (C3, C4)	Normal	Normal
IgG, IgM and IgA levels	Normal	IgA slightly decreased (0.65 g/l; ref.: 0.7-4g/l)
CRP	Normal	Normal
Sarcoidosis parameters (IL2-R, neopterine, ACE)	Negative	Negative
**Pathogens**<br>Serology for Lyme disease, lues	Negative	Negative
**Neuronal autoantibodies**		
Paraneoplastic IgG antibodies against intracellular antigens	Negative	Negative
Well-characterized neuronal IgG cell surface antibodies	Negative	Negative
Anti-MOG/AQP4-IgG antibodies	Negative	Negative
Tissue based assay on unfixed murine brain tissue (Prof. Prüss, Charité Berlin)	Slightly positive anti-nuclear antibody pattern	Moderate fine granular IgG binding against brain tissue, which is most likely to resemble anti-nuclear binding and is not expected to be pathogenic a.e. due to intracellular localization
**Cerebrospinal fluid**		
White blood cell count	1/µL (ref.: <5/µL)	1/µL (ref.: <5/µL)
Protein concentration	431 mg/L (ref.: <450 mg/L)	189 mg/L (ref.: <450 mg/L)
Albumin quotient	**7.0 (ref.: <6.0)**	2.3 (ref.: <5.7)
IgG-index	0.46 (ref.: <0.7)	0.52 (ref.: <0.7)
Oligoclonal bands in serum/CSF	Negative	Negative
Local IgG/IgA/IgM synthesis	Negative	Negative
Lactate	**1.15 mg/l (ref.: 1.5-2.1 mg/l)**	**2.58 mg/l (ref.: 1.5-2.1 mg/l)**
MRZ reaction	Negative	Negative
Well-characterized neuronal IgG cell surface antibodies	Negative	Negative
Tissue based assay on unfixed murine brain tissue (Prof. Prüss)	Slightly positive anti-nuclear antibody pattern	Moderate fine granular IgG binding against brain tissue, which is most likely to resemble anti-nuclear binding and is not expected to be pathogenic a.e. due to intracellular localization
**MRI**	**Lesion in the caudate nucleus on the right side**	**FLAIR-hyperintense lesion in the globus pallidus on the right side**
**EEG**		
Visual Assessment	No intermittent/ generalized slowing, no epileptic activity	No intermittent/ generalized slowing, no epileptic activity

Automated ICA	Alpha at 10.5Hz, clear mu-rhythms on both sides at 10.9 and 22.3Hz; spikes that can be traced back to eye muscles; **distinct frontocentral theta (suggestive of sleepiness), this leads to IRDA detections that decrease with hyperventilation**	**Lots of frontocentral theta (even peak at 5Hz)**, alpha at 10.15 and mu 12.1Hz, but no clear IRDAs
**Neuroopthalmology**	Decimal visual acuity right 1.3, visual acuity left 0.86<br>Macula/optic disc in OCT normal	Decimal visual acuity right 1.73, visual acuity left 1.37<br>**Macula in OCT: slightly flattened foveal depression on both sides**<br>Optic disc in OCT normal
**24h blood pressure measurement**	Normal	Normal
**Lactate ischemia test**	n.a.	Normal
**Stroke screening**		
24h ECG	n.a.	Normal
TTE	n.a.	Normal
Neurosongraphy (of cervical/brain) vessels	n.a.	Normal

Supplemental Table 1: Diagnostic findings in both patients (all pathological findings are marked in bold). Abbreviations: ACE, angiotensin-converting enzyme; ANAs, antinuclear antibodies; ANCAs, anti-neutrophil cytoplasmic antibodies; AQP-4, aquaporin 4 APAs, antiphospholipid antibodies; CSF, cerebrospinal fluid; CRP, C-reactive protein; ECG, electrocardiography; EEG, electroencephalography; HEp-2, Human Epithelioma-2, ICA, independent component analysis; Ig, immunoglobulin; IL2-R, interleukin 2 receptor; IRDA, intermittent rhythmic delta activity; MRI, magnetic resonance imaging; MRZ, antibody indices against measles, rubella, and varicella zoster virus; n.a., not available; ref., reference; OCI-R, Obsessive Compulsive Inventory – Revised; OCT, optical coherence tomography; SMA, smooth muscle actin; TG, thyroglobulin; TPO, thyroid peroxidase; TSH, thyroid-stimulating hormone receptor; TTE, transthoracic echocardiography; Y-BOCS, Yale-Brown Obsessive and Compulsive Scale.
